# Toward Tunable Protein‐Driven Hydrogel Lens

**DOI:** 10.1002/advs.202306862

**Published:** 2023-11-22

**Authors:** Maria Kaeek, Luai R. Khoury

**Affiliations:** ^1^ Department of Materials Science and Engineering Technion Israel Institute of Technology Haifa 32000 Israel

**Keywords:** biomaterials, optic‐biomaterials, protein nanomechanics, protein‐based materials, protein‐driven actuators

## Abstract

Despite the significant progress in protein‐based materials, creating a tunable protein‐activated hydrogel lens remains an elusive goal. This study leverages the synergistic relationship between protein structural dynamics and polymer hydrogel engineering to introduce a highly transparent protein–polymer actuator. By incorporating bovine serum albumin into polyethyleneglycol diacrylate hydrogels, the authors achieved enhanced light transmittance and conferred actuating capabilities to the hydrogel. Taking advantage of these features, a bilayer protein‐driven hydrogel lens that dynamically modifies its focal length in response to pH changes, mimicking the adaptability of the human lens, is fabricated. The lens demonstrates durability and reproducibility, highlighting its potential for repetitive applications. This integration of protein‐diverse biochemistry, folding nanomechanics, and polymer engineering opens up new avenues for harnessing the wide range of proteins to potentially propel various fields such as diagnostics, lab‐on‐chip, and deep‐tissue bio‐optics, advancing the understanding of incorporating biomaterials in the optical field.

## Graphical abstract

Despite challenges in developing a tunable protein‐activated hydrogel lens, this study introduces a transparent protein–polymer actuator by integrating bovine serum albumin into polyethylene glycol diacrylate hydrogels. This integration produces a bilayer lens that can modify its focal length based on pH variations, mirroring the adaptability of the human lens. This lens showcases resilience and holds promise for applications in diagnostics and bio‐optics, signifying progress in optical biomaterials.

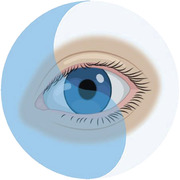

## 1 Introduction

**Figure 1:**
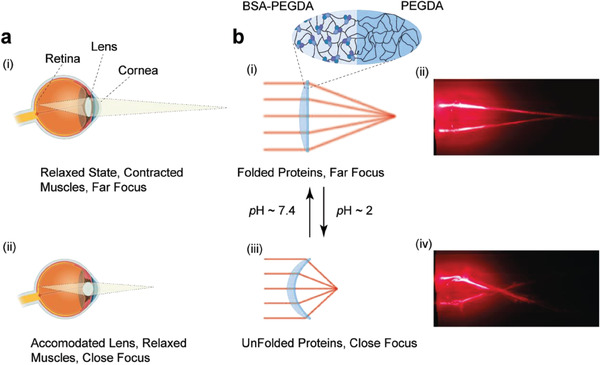
Understanding the human eye lens and exploring the tunable protein‐driven hydrogel lens. a) A cross‐sectional view illustrating the structure of the human eye lens. i) In the relaxed state, the ciliary muscles contract, causing the lens to flatten, thereby enabling long‐distant focal length for observing distant objects. ii) Accommodation occurs when the ciliary muscles relax, leading to increased lens curvature for a precise short‐range focal length for observing nearby objects. b) Protein‐driven hydrogel lens with adjustable focal length. i) A plano–convex lens created from a bilayer BSA‐PEGDA/PEGDA hydrogel in TRIS solution. ii) A close‐up image shows that the bilayer hydrogel lens effectively focuses parallel red laser rays. iii) Submerging the lens in an acidic solution (pH ≈ 2) unfolds the BSA, causing the BSA‐PEGDA hydrogel to swell, transforming the lens into a positive meniscus convex shape. iv) As a result, the focal distance of the red laser rays is shortened. The lens's focal length can be modified by reversible shape‐morphing behavior resulting from the protein structural change.

The human eye lens, made primarily from proteins, is a remarkable transparent structure that plays a vital role in vision.^[^ ^1^ ^]^ While most man‐made optical systems achieve focusing by controlling the lens displacement or using lens arrays,^[^ ^2^, ^3^, ^4^ ^]^ our human and other species' lenses can uniquely accommodate and relax their shape by using the ciliary muscle to modify and manipulate the focal length and bring objects into focus^[^ ^1^ ^]^ (**Figure** **1**a). In recent years, several approaches, such as fluid,^[^ ^5^, ^6^, ^7^ ^]^ liquid crystal,^[^ ^8^ ^]^ elastomers,^[^ ^9^ ^]^ and biopolymer‐based lenses,^[^ ^10^ ^]^ have been proposed to mimic the biomechanics of the human lens function. However, they are either complex to prepare or require external mechanical and electrical stimulation.

Most recently, globular protein‐based materials have intrigued many scientists as these materials combine unique intrinsic characteristics of proteins, such as biocompatibility, biochemical‐structural diversity, and the unique energy storage and release mechanism of protein folding transitions.^[^ ^11^, ^12^, ^13^ ^]^ Many strategies have been suggested to bring them to the forefront of the biomaterials field, such as using their inherent characteristics to manipulate their stiffness and microstructure,^[^ ^13^, ^14^, ^15^, ^16^, ^17^, ^18^, ^19^, ^20^, ^21^ ^]^ designing protein‐based materials for shape‐memory^[^ ^22^, ^23^ ^]^ and morphing behavior,^[^ ^24^ ^]^ and self‐healing application.^[^ ^25^ ^]^ While there have been significant advancements in protein‐based materials, the combination of transparency and shape‐memory behavior in a single material remains challenging.

Few methods have been proposed to generate transparent protein‐based materials in recent years. For example, hydrogels made of egg white solution under unfriendly‐to‐protein alkaline conditions (NaOH, pH≈14) based on physical crosslinking between protein molecules showed a transparency of almost 99.8%.^[^ ^26^, ^27^ ^]^ Recently, a study investigating the gelation of a mixture of pigeon egg white with ionic surfactants at boiling temperatures (≈90 °C) resulted in a transparent hydrogel with 99% light transmittance.^[^ ^28^ ^]^ These strategies use experimental processes such as alkyl hydrolysis, high temperatures, and time‐consuming reactions,^[^ ^29^ ^]^ which lead to structural alteration and aggregation and conceal the potential of protein‐folded structure and functionality.^[^ ^30^ ^]^ An intriguing example is a tunable transparent protein‐based lens, which uses protein nanomechanics to mimic focal accommodation and relaxation in a human lens.

Here, we leverage the structural folding transitions of bovine serum albumin (BSA)^[^ ^14^, ^21^, ^22^ ^]^ along with the transparent properties of polyethyleneglycol diacrylate (PEGDA) hydrogel transparency^[^ ^31^, ^32^ ^]^ to introduce, for the first time, a biocompatible transparent shape‐memory BSA‐PEGDA lens with autonomous focusing capabilities (Figure 1b). Our system's fundamental design involves a bilayer hydrogel actuator composed of two layers: an active layer and a passive layer. These layers incorporate BSA‐PEGDA and PEGDA hydrogels, respectively. The hydrogel layers were formed through a simple UV photocrosslinking reaction with lithium phenyl‐2,4,6‐trimethyl‐benzoyl phosphinate (LAP) photoinitiator without affecting the protein secondary structure. The sensitivity of BSA structural nanomechanics to acidic (pH ≈ 2) and neutral pH environments and the significant difference in swelling between the two layers exert a big enough force to actuate the protein‐driven lens from plano–convex to the convex–concave lens and hence tune the focal length from *f* = 63 ± 2 to 23 ± 2 mm (Figure 1b). Another striking result is our ability to manipulate a diverse range of focal lengths, including the ability to mimic a human lens's focal length range (58 ± 2 to 41 ± 0.5 mm) through our approach. Our development, which incorporates proteins as the building blocks of biocompatible lenses, has the potential to open up new possibilities for the design and fabrication of optical systems.

## 2 Results

### 2.1 Synthesis and Characterization of BSA‐PEGDA Hydrogels

**Figure 2:**
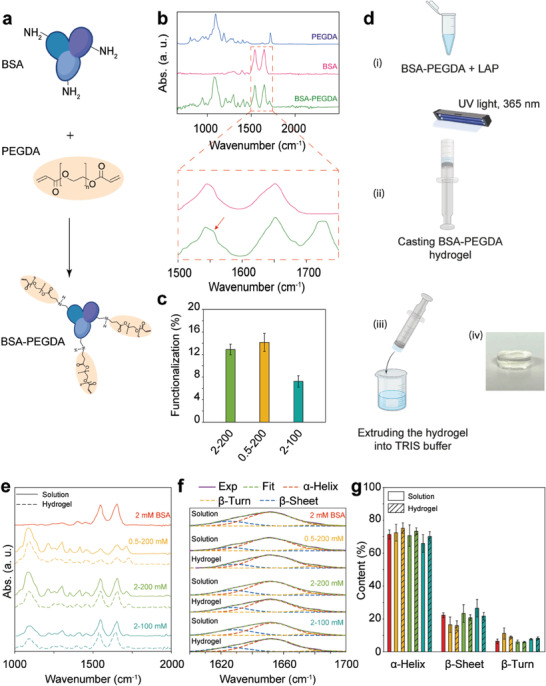
Photocurable BSA‐PEGDA mixture: formation, characterization, and hydrogel synthesis. a) Formation of a photocurable BSA‐PEGDA mixture through the aza‐Michael addition reaction between lysine residues on BSA and acrylates on PEGDA, leading to the formation of lysine‐functionalized BSA‐PEGDA solution. b) ATR‐FTIR spectra of 200 mm PEGDA solution, 2 mm BSA solution, and 2–200 mm BSA‐PEGDA solution at RT. Inset: Zoomed‐in view of the amide I and II peaks in the 2 mm BSA solution and 2–200 BSA‐PEGDA mixture. The red arrow indicates a peak at 1540 cm^−1^, corresponding to secondary amines resulting from the aza‐Michael addition reaction between BSA lysine and PEGDA acrylates. c) Percentage of lysine functionalization of BSA by PEGDA acrylates at different concentration ratios. d) Synthesis of BSA‐PEGDA hydrogel: i) The BSA‐PEGDA mixture was mixed with LAP (19:1 v/v), degassed to remove air bubbles, and poured into a casting mold. ii) The mixture was exposed to UV light at 365 nm (5.4 mW cm^−2^) for 1 min at RT, initiating radical polymerization. iii) The hydrogel was then transferred to the TRIS buffer. iv) Close‐up image of a transparent BSA‐PEGDA hydrogel sample. e) ATR‐FTIR spectra of BSA solution, BSA‐PEGDA mixtures, and hydrogel samples at various ratios. f) Determination of BSA's secondary structure through Fourier deconvolution of the amide I band (1600–1700 cm^−1^) for BSA domains before and after gelation. The deconvolution revealed three main secondary structures: intramolecular β‐sheet, α‐helix, and β‐turn. g) Calculation of the conformational content of BSA's secondary structure pre‐ and post‐gelation by measuring the area under each peak. Error bars indicate the standard deviation in all measurements.

BSA is a widely studied, abundant, and comparatively cheap protein with diverse applications due to its structural diversity and conformational flexibility.^[^ ^33^, ^34^, ^35^, ^36^ ^]^ However, to harness the structural folding transitions of BSA together with PEGDA transparency,^[^ ^31^, ^32^ ^]^ it must be covalently integrated into the PEGDA network. PEGDA with low molecular weight, like PEGDA700, possesses a lower viscosity than high molecular‐weight PEGDA, such as PEGDA 5000 and PEGDA 10 000, resulting in improved ease of handling and processing. Furthermore, low molecular weight PEGDA exhibits a moderate swelling behavior,^[^ ^37^ ^]^ which has a minimal impact on the functioning of the bilayer actuating system. Therefore, we employed the aza‐Michael addition reaction between the lysine residues on the BSA surface and the acrylate of PEGDA700 to generate BSA‐PEGDA complexes (**Figure** **2**a).^[^ ^38^ ^]^ The successful modification of lysine was confirmed by the appearance of a peak around ≈1540 cm^−1^ in the attenuated total reflectance‐Fourier transform infrared spectroscopy (ATR‐FTIR) recordings corresponding to the secondary amines (Figure 2b). Furthermore, we examined how the concentration ratio of PEGDA and BSA influenced the degree of lysine residue functionalization, employing the 2,4,6‐trinitrobenzene sulfonate (TNBS) assay (Figure S1, Supporting Information). Our results revealed that reducing the PEGDA concentration from 200 to 100 mm in 2–200 and 2–100 BSA‐PEGDA complexes decreased the lysine functionalization from 13 ± 1% to 7 ± 1%. However, the modification percentage had no noticeable impact when the BSA concentration decreased from 2 to 0.5 mm while maintaining the PEGDA concentration at 200 mm (Figure 2c). Subsequently, we proceeded to synthesize BSA‐PEGDA hydrogel samples by mixing a BSA‐PEGDA complex solution at different molar ratios (0.5–200, 2–200, and 2–100 mm) with 50 mm LAP in a volume ratio of 19:1, respectively. The resulting mixture was poured into a cut‐head syringe to achieve cylindrical shape samples with precise dimensions. Then, the solution was exposed to UV light (5.4 mW cm^−2^) for 1 min at room temperature (RT). The hydrogel was then extruded into TRIS solution (20 mm Tris, 150 mm NaCl, pH≈7.4) for equilibration (Figure 2d). Since protein structure is important in developing shape‐memory protein‐based materials, we employed ATR‐FTIR analysis to characterize native 2 mm BSA solution, BSA‐PEGDA complex solutions, and hydrogel samples (Figure 2e). By deconvoluting the amide I peak (1600–1700 cm^−1^) of the solution and hydrogel samples, we observed three main structures β‐sheet (1610–1630 cm^−1^), α‐helix (1648–1660 cm^−1^), and β‐turn (1660–1689 cm^−1^), representing the secondary conformation of BSA protein (Figure 2f). Moreover, the area under each fitting curve was calculated, comprising ≈20% intramolecular  β‐sheet, ≈70% α‐helix, and 10% β‐turn for all samples, which agrees with previously published results^[^ ^21^, ^39^ ^]^ (Figure 2g).

### 2.2 Optical Transparency and Actuating Behavior of BSA‐PEGDA Hydrogels

**Figure 3:**
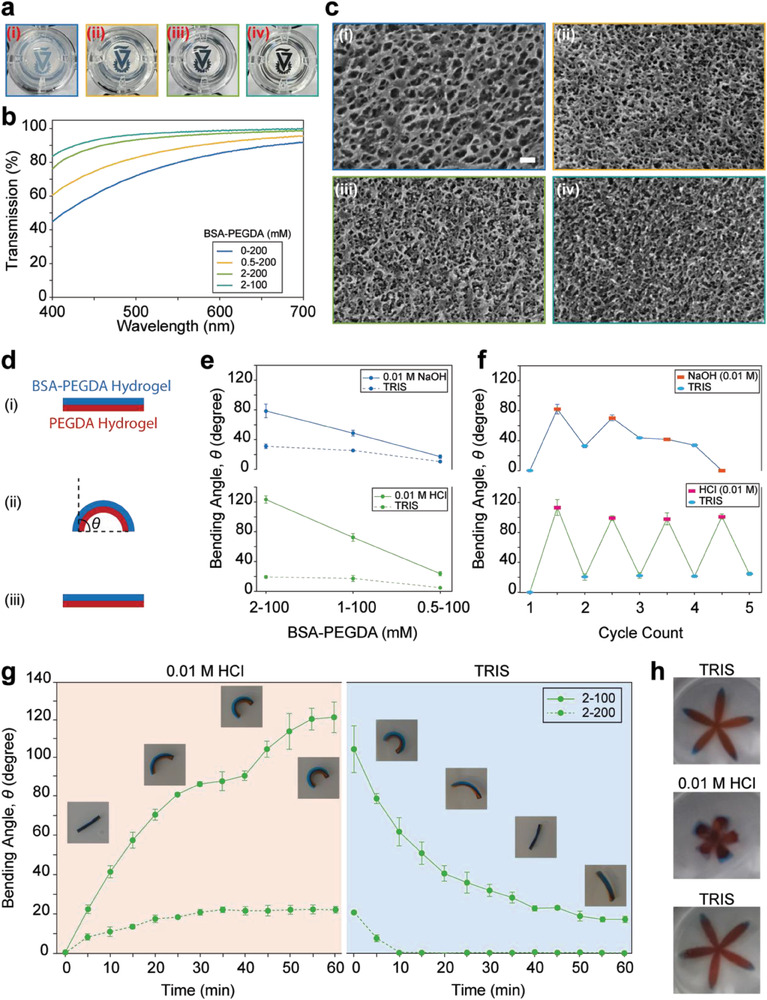
Exploring transparency, bending angles, and morphing kinetics in BSA‐PEGDA hydrogels. a) Close‐up images of BSA‐PEGDA hydrogels with various concentration ratios were placed over the Technion logo, showcasing their transparency. i) 0‐200, ii) 0.5‐200, iii) 2‐200, and iv) 2‐100 mM. b) Measurement of the light transmission spectra in BSA‐PEGDA hydrogels. c) Cryo‐SEM images of BSA‐PEGDA hydrogels at different concentration ratios: i) 0–200, ii) 0.5–200, iii) 2–200, and iv) 2–100 mm. d) Schematic representation of the actuating behavior of the unimorph BSA‐PEGDA/PEGDA bilayer hydrogel: i) Bilayer hydrogel in TRIS (pH ≈ 7.4). ii) Immersion in 0.01 m NaOH (pH ≈ 12) or 0.01 m HCl (pH ≈ 2) triggers BSA unfolding, causing the BSA‐PEGDA layer to swell and bend toward the PEGDA layer. The bending angle, *θ*, represents the deviation from the original linear position. iii) Recovery of the bilayer hydrogel actuator to its initial shape. e) Measurement of bending angles in bilayer hydrogels for different BSA concentrations. Each sample was soaked in the respective solution for 1 h. f) Variation in the bending angle of the bilayer hydrogel system after alternating immersion in 0.01 m NaOH or HCl and TRIS (pH ≈ 7.4) solutions for 1 h each over five successive cycles. The graph demonstrates a complete cyclic behavior of the bilayer hydrogel in an acidic environment, while the sample in the basic solution failed after the third cycle. g) Investigation of the effect of PEGDA concentration on bilayer actuating behavior. The left side of the graph (orange background) depicts the morphing kinetics of the bilayer hydrogel system after 1 h immersion in 0.01 m HCl (pH ≈ 2) at RT. The right side (blue background) shows the recovery of the bilayer hydrogel system when immersed in TRIS (pH ≈ 7.4). Images illustrate the bending behavior of the 2–100 bilayer hydrogel system in different solutions. h) A flower‐like bilayer hydrogel system under acidic conditions. The petals of the flower close (upward) when immersed in 0.01 m HCl and reopen in TRIS. Error bars represent the standard deviation in all measurements.

PEGDA hydrogels are known for their transparency.^[^ ^31^, ^32^ ^]^ However, incorporating BSA into these hydrogels can achieve the desired optical properties in BSA‐PEGDA samples. Our observation of the hydrogel samples (**Figure** **3**a) revealed that 0–200 mm hydrogel without BSA exhibited slight opacity, with a light transmission lower than 80% in the blue‐to‐green range of 450–550 nm (Figure 3b). Nonetheless, incorporating BSA in the PEGDA network improved light transmission. For instance, as we increased the protein concentration to 0.5 mm, the BSA‐PEGDA‐based hydrogel samples became less opaque (Figure 3a), enhancing transparency. The hydrogel displayed high clarity in the 2–200 sample, and the transmission reached almost ≈95% in the visible range (400–700 nm) (Figure 3b). Decreasing the PEGDA concentration to 100 mm, the 2–100 hydrogel sample showed a slight improvement in the light transmission, particularly in the short wavelength range, the hydrogel exhibited exceptional transparency (>95%). To gain insights into these findings, we employed cryo‐scanning electron microscopy (cryo‐SEM) to examine the microstructure of the BSA‐PEGDA hydrogel samples and evaluate the impact of BSA integration on the PEGDA hydrogel. The 0–200 mm hydrogel displayed a highly porous microstructure with hundreds of nanometer pore sizes. However, upon integrating BSA in the PEGDA network, the 0.5–200 revealed a denser arrangement with a higher presence of lamellar morphology and smaller pore sizes than the PEGDA sample. While increasing the BSA to 2 mm in 2–200 mm hydrogel, the lamellar microstructures decreased, and the pore density increased.

On the other hand, in the 2–100 hydrogel, the microstructure exhibited more homogeneously fibrous porous arrangements with tens of nanometers pore sizes (<20 nm) (Figure 3c). These observations align with the swelling behavior of the hydrogel samples, as indicated in Figure S2 (Supporting Information). These results can be ascribed to the increase in the crosslinking density due to BSA incorporation, leading to the formation of micro‐ and nano‐sized pores, which acts as light‐scattering spots and affects the transparency.^[^ ^31^, ^40^, ^41^, ^42^ ^]^

After considering the excellent transparency exhibited by the 2–100 hydrogel sample and the notable disparity in swelling ratio and stiffness (Figures S2–S4, Supporting Information) compared to the 2–220 and 0–200 hydrogels, we decided to utilize the 2–100 hydrogel samples for the subsequent bilayer hydrogel actuator studies. Thus, an unimorph bilayer hydrogel actuator was fabricated from a 2–100 complex as an active layer and 0–200 as the passive layer in a 1:1 volume ratio, which would exhibit a distinctive bending angle^[^ ^23^, ^43^ ^]^ (Figure 3d and Figure S5, Supporting Information). Furthermore, considering the sensitivity of BSA's conformational structure to acidic and basic environments,^[^ ^33^, ^34^, ^35^ ^]^ the bilayer hydrogel actuator bends toward the passive layer in 0.01 m HCl (pH ≈ 2) and NaOH (pH ≈ 12). Subsequently, the actuator would recover its original shape when placed in a TRIS solution (Figure 3d). First, we proceeded to investigate the impact of protein concentration on the actuating behavior of the hydrogel system. We discovered that as the protein concentration increased while maintaining a constant PEGDA concentration (100 mm), the bilayer hydrogel exhibited a more significant bending response in both acidic and basic conditions.

Conversely, when the protein concentration was decreased, the bending behavior of the hydrogel system almost disappeared (Figure 3e). These findings can be attributed to the swelling and deswelling behavior driven by the protein‐folding nanomechanics within the active layer of the hydrogel. In addition, these results highlight the importance of incorporating the BSA in the bilayer protein‐based hydrogel system to manipulate the morphing kinetics of the system.

Furthermore, we investigated the impact of sequential immersion of the bilayer system in acidic and alkaline environments on the actuating behavior of the hydrogel. The hydrogel samples were alternatively immersed in NaOH or HCl solutions, with TRIS serving as an intermediate step. This cyclic process was repeated five times, for 1 h each. The bending angles of the hydrogel samples immersed in HCl exhibited successful reversibility. However, the bending angle of the hydrogel samples immersed in NaOH gradually decreased with each subsequent soaking cycle. Eventually, after the third cycle, the hydrogel samples ceased to undergo morphing, indicating a loss of reversibility (Figure 3f). These results highlight the detrimental effect of alkaline conditions, which disrupt the native alpha‐helix structure that makes up 70% of the BSA structure (Figure 2g), and lead to irreversible damage to S─S bridges through oxidation of S^−^. Conversely, in an acidic environment, the denaturing effect on the BSA structure is relatively mild, with less involvement of S─S bonds, which helps preserve their integrity.^[^ ^34^, ^35^, ^36^ ^]^ Based on these findings, all subsequent morphing kinetics experiments were conducted using 0.01 m HCl solutions.

Next, we investigated the impact of PEGDA concentration on the bending angle behavior of the bilayer hydrogel actuator over 1 h (Figure 3g and Movie S1, Supporting Information). Increasing the PEGDA concentration from 100 to 200 mm made more lysine residues functionalized with PEGDA (Figure 1c). This resulted in a higher crosslinking density, greater immobilization of BSA domains, reduced swelling ratio, and increased stiffness (Figures S2–S4, Supporting Information). Therefore, when the 2–100 bilayer system was immersed in 0.01 m HCl, it exhibited a higher bending angle than the 2–200 hydrogel actuator (120 ± 8° vs 22 ± 2°). Upon immersing the systems in the TRIS solution, the 2–100 and 2–200 recovered to 80% and 100% of their initial shape, respectively. In addition, the 2–200 exhibited a faster recovery than the 2–100 actuator. These observations provide further evidence highlighting the significance of protein (un)folding nanomechanics and its influence on the tunability of actuating behavior in the bilayer hydrogel system. Additionally, the latter behavior and the feasible hydrogel preparation process allowed us to design a flower‐like shape actuator that can close and open its petals in acidic and neutral environments (Figure 3h; Figure S5 and Movie S2, Supporting Information).

### 2.3 Tunable Protein‐Driven Hydrogel Lens

**Figure 4:**
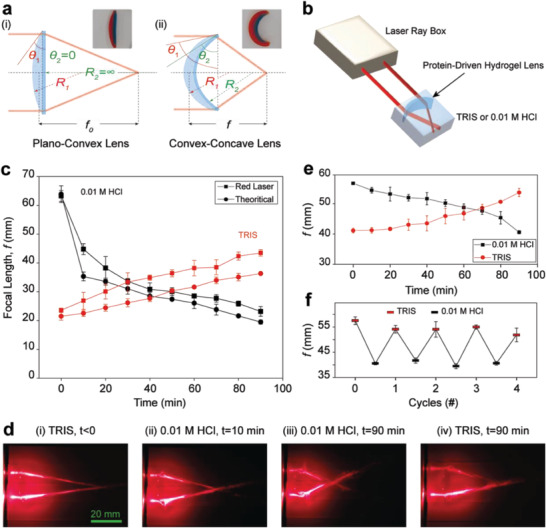
Design and characterization of protein‐driven hydrogel lenses for adjustable focal length. a) i) Schematic representation of the bilayer BSA‐PEGDA/PEGDA hydrogel lens, designed as a plano–convex lens. Inset: Close‐up view of the bilayer hydrogel sample immersed in TRIS buffer. The BSA‐PEGDA layer is indicated in red dye, while the PEGDA layer is colored with blue dye. ii) Diagram illustrating the structure of the convex–concave bilayer hydrogel lens. Inset: Image of the bilayer hydrogel sample after immersion in HCl buffer. The curvature radii *R*_1_ and *R*_2_ were derived from bending angles and arc length measurements. b) Visual rendering of the experimental setup with a protein‐driven hydrogel lens perpendicular to the laser ray box. The hydrogel lens undergoes sequential immersion in TRIS and 0.01 m HCl to induce shape‐morphing, transforming it from a plano–convex to a convex–concave lens, thereby adjusting the focal length. c) Comparison of calculated and measured focal lengths for the 2–100/0–200 hydrogel lens after 90 min of immersion in 0.01 m HCl (pH ≈ 2) at RT and during recovery when immersed in TRIS. d) Close‐up images demonstrate the focal length variation in HCl and TRIS solutions. i) A plano–convex protein‐driven hydrogel lens in TRIS. ii) After 10 min in HCl, the hydrogel lens displays shape‐morphing from plano–convex to convex–concave, reducing the focal length. iii) The hydrogel system after 90 min in HCl solution, showing a significant change in focal length. iv) After being immersed in TRIS for 90 min, the lens returned to its initial convex–concave shape, with a focal length similar to that in (ii). e) Replication of the human lens focal length achieved by substituting the 0–200 layer with a 2–200 hydrogel. f) Recovery of the bilayer hydrogel's focal length after undergoing alternate treatments with HCl and TRIS for five cycles.

The remarkable capability of bilayer protein–polymer hydrogels to undergo shape changes in response to external triggers, such as pH, combined with their exceptional optical transparency, has paved the way for designing protein‐driven hydrogel lenses with tunable focal lengths. Initially, we used the bilayer actuator concept to fabricate a plano–convex bilayer hydrogel lens (**Figure** **4**a(i)). With water constituting most of the hydrogel (≈90%) and immersed in a buffer, the measured refractive index was about 1.34. We derived the curvature's radius using bending angle and arc length measurements (Figures S7–S9, Supporting Information) and subsequently determined the focal length using the lens maker equation to be *f*_0, calculated_ = 64 ± 3 mm (Figure 4a). When aligning the lens perpendicular to parallel dual red laser beams (Figure 4b), it effectively converged the rays at a measured focal length of *f*_0, measured_ = 63 ± 2 mm (Figure 4c,d). Subsequently, submerging the lens in an acidic solution (0.01 m HCl) induced a transformation into a convex–concave lens (Figure 4a(ii)). This alteration resulted from the disparity in swelling caused by protein unfolding, which decreased the curvature radii and reduced the measured focal length to *f* = 23 ± 2 mm over nearly 2 h (Figure 4c,d). Immersing the lens back into the TRIS solution led to a decrease in curvature and an increase in focal length up to 44 ± 3 mm (Figure 4c,d and Movie S3, Supporting Information). To control the focal length tunability, we replaced the 0–200 mm BSA‐PEGDA hydrogel with 2–200 mm BSA‐PEGDA hydrogel, which showed high transparency (Figure 3b) and impressive recovery after being moved from HCl to TRIS (Figure 3g). We can see that we successfully manipulated the focal length range between 58 ± 2 to 41 ± 0.5 mm and, most importantly, achieved a full recovery (Figure 4e). Additionally, we examined the lens's reversibility and found that our lens successfully recovered its shape after each cycle (Figure 4f).

## 3 Discussion

Several approaches have been devoted to improving the transparency of hydrogel lenses. A recent study developed hydrogel contact lenses using polyvinyl alcohol and polyvinylpyrrolidone. While they integrated silver nanoparticles to refine optical properties and mitigate chromatic aberration, these nanoparticles presented challenges, particularly in aggregation, which resulted in uneven distribution and potential visual distortions.^[^ ^44^ ^]^ Similarly, another methodology introduced transparent hydrogel lenses synthesized from acrylic acid and *N*,*N*‐dimethylacrylicamide. The enhancement in transparency was attributed to in situ polymerization in the presence of aluminum hydroxide nanoparticles through ion coordination. Yet, this non‐covalent crosslinking presented challenges, such as a potential leakage of nanoparticles, which might adversely impact the hydrogel's mechanical and optical properties. Ensuring uniform nanoparticle dispersion within the hydrogel was complex, leading to inconsistencies in optical characteristics. Compounding these technical challenges was the overarching concern regarding the long‐term biocompatibility of these nanoparticles.^[^ ^45^ ^]^ Another study produced hydrogel lenses using a combination of a hydrophobic silicone monomer and a hydrophilic monomer, *N*,*N*‐dimethylacetamide. To enhance transparency, additives like holmium (III) oxide, europium (III) oxide, and aluminum oxide were incorporated, reducing surface roughness and improving wettability. However, using multiple additives made optimizing the lens properties cumbersome.^[^ ^46^ ^]^ Standing distinct from these methodologies, our approach relied on fusing two biocompatible materials, BSA and PEGDA, to form a covalently bonded network through a straightforward and efficient method to produce a hydrogel lens.

While numerous studies have used diverse protein biochemical structures to embed them into polymer hydrogels,^[^ ^25^, ^38^, ^47^ ^]^ a unique feature of our system is that we used BSA to manipulate the microstructure of PEGDA hydrogels to improve their transparency up to ≈95% without compromising the protein secondary structure.^[^ ^26^, ^28^, ^29^ ^]^ In addition, our hydrogel transparency is preserved even under acidic conditions (pH ≈ 2), as the mild denaturing of the BSA molecules (Figures 3f and 4f) tends to yield a consistent and evenly dispersed arrangement of the denatured BSA molecules, increasing the swelling behavior, and contributing to its transparency.^[^ ^16^ ^]^ Additionally, BSA functionalization with PEGDA acted as a spacer between BSA molecules in the hybrid BSA‐PEGDA network. This effectively reduces BSA aggregations during structural alternations, preventing the hydrogel from becoming opaque. Another attractive feature is that we covalently incorporated a small quantity of BSA into the PEGDA network and utilized BSA structural dynamics with the light transmittance characteristic of PEGDA to design transparent tunable protein‐driven optical actuators for the first time.

Various methods have been suggested for forming tunable lenses broadly,^[^ ^3^, ^5^, ^6^, ^7^, ^9^, ^48^, ^49^ ^]^ with a particular emphasis on lenses made from hydrogel materials^[^ ^10^ ^]^ with fast response time. While our system is much slower, we can enhance its response time by manipulating protein structure and (un)folding nanomechanics response time using protein engineering technologies or by employing 3D printing technologies to reduce the protein‐driven lens system's size.^[^ ^50^, ^51^ ^]^ However, our system does not require complex external triggering systems; instead, we utilize physiological‐like conditions, such as in the stomach (pH ≈ 2) and small intestine (pH ≈ 7.4), to adjust the focal length or to morph the shape of a biocompatible imperceptible actuator, which can be used for drug delivery and sensing applications.^[^ ^52^ ^]^ Furthermore, compared to current systems,^[^ ^10^ ^]^ we manipulated our protein‐driven lens to attain a focal length range similar to the human lens (61.4 to 43.5 mm).^[^ ^1^ ^]^

While the primary trigger for our lenses is acidic pH, their ability to adapt their shape in response to pH changes introduces both versatility and precision. This attribute amplifies their transformative potential across various scientific disciplines. For example, photodynamic therapy (PDT) uses light‐sensitive compounds to kill cancer cells in cancer treatment. pH‐sensitive lenses can precisely control the light delivery in PDT. The lens can adjust its shape in response to the acidic pH environment within the tumor,^[^ ^53^ ^]^ ensuring targeted and efficient light activation of the photosensitizing agents, thus enhancing the therapeutic outcome.^[^ ^54^ ^]^ In addition, lenses responsive to pH changes can have a pivotal impact on optogenetics, a biological technique utilizing light to manipulate cells within living tissues. pH‐sensitive lenses can modulate the light intensity and focus in optogenetic experiments.^[^ ^55^ ^]^

Beyond biological applications, pH‐sensitive lenses can be integrated into microfluidic systems. The change in lens shape due to pH variations can be used to control the flow of fluids within microchannels. This can enable the development of microfluidic devices where chemical reactions or analyses are triggered by specific pH conditions, leading to applications in lab‐on‐a‐chip technology and biochemical assays.^[^ ^56^ ^]^

Moreover, pH‐sensitive lenses can be utilized in medical imaging, particularly in endoscopes and imaging devices. This can result in clearer and more detailed imaging of tissues with varying pH environments, aiding diagnostics and research development.^[^ ^57^, ^58^, ^59^, ^60^ ^]^

In conclusion, the present approach exhibits remarkable adaptability, primarily attributed to the extensive protein library available. This approach can be applied to numerous other transparent protein‐driven systems that respond to diverse stimuli, including light, temperature, and biomolecules, through appropriate engineering and structural modifications of protein and polymers. This advancement could propel the field of protein‐based materials into the forefront of optics, facilitating the development of tunable bio‐lens arrays for a wide range of applications such as lab‐on‐chip technology, diagnostics, and deep tissue bio‐optics.

## 4 Experimental Section

### Protein‐Based Hydrogel Synthesis

BSA and PEGDA (Mn = 700 g mol^−1^) were mixed at vigorous stirring for 24 h at RT in TRIS buffer solution (20 mm Tris, 150 mm NaCl, pH ≈ 7.4) to form BSA‐PEGDA mixture at different concentration ratios (0–200, 0.5–200, 2–200, and 2–100 mm). Then, to prepare the BSA‐PEGDA hydrogel sample, the mixture was mixed with a photoinitiator LAP (50 mm in TRIS, pH ≈ 7.4) at a 19:1 v/v ratio. After that, the mixture was centrifuged to remove air bubbles and loaded into a casting mold or a transparent tube. Then, the mixture was moved under UV light at 365 nm (5.4 mW cm^−2^) for 1 min at a 3 cm distance at RT to induce LAP photocleavage and the radical‐initiated chain polymerization process between the PEGDA acrylates. Once the hydrogel sample was obtained, it was extruded and subjected to three washes using TRIS buffer to remove LAP and intermediate products.

### ATR‐FTIR

ATR‐FTIR spectra of each 2 mm BSA solution and BSA‐PEGDA (0.5‐200, 2–200, and 2–100 mm) mixtures and hydrogel samples were recorded using Nicolet iS50 FTIR via ATR mode equipped with a round Diamond (Type IIa crystal). Sixteen scans were recorded for each sample with a nominal resolution of 8 cm^−1^. The different conformations of the three main secondary structures of BSA for both in solutions and hydrogels, including intramolecular β‐sheets (1610–1630 cm^−1^), α ‐helix (1648–1660 cm^−1^), and β‐turns (1660–1689 cm^−1^), were analyzed by the spectral deconvolution of the amide I band (1600 to 1700 cm^−1^) using OMNIC FTIR Software.

### TNBS Assay

A triplicate of 2 mm BSA and BSA‐PEGDA (0.5–200, 2–200, and 2–100 mm) solutions was prepared. The mixtures were purified via dialysis against DDH_2_O for 4 days at RT. After that, the dialyzed solutions were freeze‐dried at −79 °C using liquid nitrogen and then moved to a lyophilizer at 0.016 mBar to obtain BSA and BSA‐PEGDA powders. Afterward, each powder was dissolved in carbonate–bicarbonate buffer (34 mm sodium bicarbonate, 16 mm sodium carbonate [anhydrous], pH ≈ 9.6) to a final concentration of 200 µg mL^−1^. Then, 0.25 mL of 0.01% w/v solution of TNBS was added to 0.5 mL of each solution, and the samples were incubated at 37 °C for 2 h. After that, 0.25 mL of 10% sodium dodecyl sulfate and 0.125 mL of 1 N HCl were added to each sample to quench the reaction. The absorbance of each solution was measured at 342 nm using UV–visible spectrophotometry. The percentage of lysine functionalization of BSA with PEGDA acrylates was then calculated as follows.

(1) $$\mathrm{functionalization}\;\left(\%\right)=\frac{A_{\mathrm{BSA}}-A_{\mathrm{BSA}+\mathrm{PEGDA}}}{A_{\mathrm{BSA}}}$$

### Swelling Ratio Measurements

BSA‐PEGDA hydrogels (0–200, 0.5–200, 2–200, and 2–100 mm) were prepared and immersed in TRIS for 24 h at 4 °C to ensure complete swelling. Afterward, the hydrogels were removed from the TRIS, and the excess solution was removed using filter paper. Then, the hydrogels were weighed by analytical balances to obtain their wet weight (*W*_wet_). Afterward, the hydrogel samples were washed three times with DDH_2_O at RT and freeze‐dried. After 24 h, the dry weight (*W*_dry_) was obtained by weighing the dry hydrogels. The swelling ratio (SR) was calculated using the following equation.

(2) $$S.R.\left(\%\right)=\left(W_{\mathrm{wet}}-W_{\mathrm{dry}}\right)/W_{\mathrm{dry}}*\times 100$$

### Optical Transmission Measurements

From each BSA‐PEGDA hydrogel mixture (0–200, 0.5–200, 2–200, and 2–100 mm), 200 µL was pipetted into a 96‐well microplate and cured with UV light. Afterward, the absorbance (A) of the hydrogel samples was measured using UV–visible spectrophotometry (Cary 100) with a wavelength range of 400–700 nm. To obtain the optical transmission (T) spectrum of the hydrogel samples, the transmission percentage was calculated by using the Beer–Lambert law‐based equation.

(3) $$T\;\left(\%\right)\;=10^{2-A}$$

### Cryo‐SEM

BSA‐PEGDA hydrogels (0–200, 0.5–200, 2–200, and 2–100 mm) were washed three times with DDH_2_O at RT. The hydrogel samples were inserted between two aluminum discs (3 mm in diameter, 25 µm thick each) and then cryo‐immobilized in a high‐pressure freezing device (EM ICE, Leica). The samples were mounted on a holder under liquid nitrogen in a loading station (EM VCM, Leica) and transferred under cryogenic conditions (EM VCT500, Leica) to a sample preparation freeze fracture device (EM ACE900, Leica). The samples were fractured by a rapid stroke of a cryogenically cooled knife to expose the inner part between the discs. Afterward, the samples were etched (−100 °C, for 10 min) to sublimate ice from the sample surface and then coated with 3 nm carbon. A secondary electron in‐lens detector was used to image the samples (Gemini SEM (Zeiss)‐operating temperature of −120 °C). The measurements were done at the Ilse Katz Institute for Nanoscale Science and Technology at the Ben‐Gurion University of the Negev, Beer Sheva, Israel.

### Observation of Shape Morphing under Acidic and Basic Environments

A rectangular (10 mm, 4 mm, 4 mm, *L* × *W* × *H*) and flower shape molds were printed using the ANYCUBIC Photon MONO X 6K 3D Printer. To obtain the casting mold for these defined shapes, Dragon Skin silicones 20 were poured into the templates. To enhance the visibility of the 2–200 and 2–100 samples, red and blue dyes were added to the PEGDA and BSA‐PEGDA hydrogel mixtures, each in a quantity of 1 µL.

The PEGDA/ BSA‐PEGDA bilayer hydrogel systems (1:1 v/v) were prepared as follows: Initially, a 65 µL mixture of 0–200 mm PEGDA hydrogel was poured into the rectangular mold and cured under UV light at 365 nm (5.4 mW cm^−2^) for 1 min at RT. Subsequently, a second layer of BSA‐PEGDA (2‐100 or 2–200 mm) mixture (65 µL) was added to the first layer and cured using the same setup. After extruding the samples from the mold, they were immersed in TRIS buffer at RT for 5 min to achieve equilibrium.

To observe the kinetics of shape morphing in the unimorph bilayer hydrogel system, the hydrogel samples were soaked in 0.01 m NaOH (pH = 12) or 0.01 m HCl (pH = 2) for 1 h at RT. The entire soaking process was recorded using a time‐lapse video, capturing one picture every 5 s. The bending angle *θ*, representing the deviation from the linear position, was measured every 5 min by analyzing the time‐lapse video images using ImageJ. Subsequently, the bilayer hydrogels were immersed in TRIS buffer for 1 h at RT to observe the shape recovery toward the linear configuration. The bending angle was also calculated from the time‐lapse images.

### Protein‐Driven Hydrogel Formation

The hydrogel lens with a plano‐convex shape (20 mm diameter, 4 mm center thickness, and 1 mm edge thickness) was fabricated through 3D printing using the ANYCUBIC Photon MONO X 6K 3D Printer. To create a negative mold of the bilayer hydrogel lens, Dragon Skin silicones 20 were poured into the template. Following this, a mixture of 2–100 mm was added (185 µL) to fill the curved part of the mold's hemispherical space and then cured under UV light at 365 nm (5.4 mW cm^−2^) for 1 min at RT. Subsequently, a second layer of 0–200 or 2–200 hydrogel mixture (BSA‐PEGDA) was added (100 µL) on top of the first layer, creating the plano space, and cured using the same UV setup. The resulting plano–convex lens hydrogels were removed from the mold and soaked in TRIS buffer (pH 7.4) at RT to achieve equilibrium.

### Tuning Focal Length Using Protein‐Driven Hydrogel Lens

An experimental setup involved placing a hydrogel lens perpendicular to a monochromatic red laser ray box that emitted two red laser beams (each with a diameter of 1 mm), with the convex side facing the laser source. Initially, the hydrogel lens was immersed in a TRIS solution. The focal length (*f*_measured_) was then determined by measuring the distance from the lens's center to the laser beams' intersection point. Subsequently, the TRIS solution was replaced with 0.01 m HCl, and the focal length was measured every 10 min for 1.5 h.

Following this, a bilayer hydrogel lens sample was immersed in TRIS solution for the same duration to observe the shape recovery of the positive meniscus lens toward the plano–convex configuration. The focal length was measured using the same method as previously described.

The changes in bending angles *θ*1, *θ*2, and arc lengths *L*1 and *L*2 of the hydrogel lens were measured every 10 min by analyzing the time‐lapse video images using ImageJ to compare the measured focal lengths with the theoretical values. The radii *R*_1_ and *R*_2_ corresponding to the front and back surfaces of the lens were then calculated using the equation

(4) $$R_{1/2}=\frac{180^{\circ }}{\pi }\frac{L_{1/2}\;}{2\;\theta_{1/2}}$$

Subsequently, the theoretical focal length *f*_theoritical_ was calculated using the lens maker equation.

(5) $$\;\frac{1}{f_{\mathrm{theoritical}}}=\left(n-1\right)\left(\frac{1}{R_{1}}-\frac{1}{R_{2}}\right)$$

where *n*  =  1.34 represents the average refractive index of the hydrogel layers determined by Schmidt and Haensch (Abbe model AR12) refractometer.

## Conflict of Interest

The authors declare no conflict of interest.

## Author Contributions

L.R.K. designed the research. M.K. performed the experiments. M.K. analyzed most of the data. L.R.K. wrote the manuscript, and all authors reviewed the manuscript.

## Supporting information

Supporting Information

Supplemental Movie 1

Supplemental Movie 2

Supplemental Movie 3

## Data Availability

The data that support the findings of this study are available from the corresponding author upon reasonable request.
