# Supplementary material for: Toward Tunable Protein‐Driven Hydrogel Lens
Source: Adv Sci (Weinh). 2023 Nov 22;10(36):2306862. doi: 10.1002/advs.202306862 (PMC10754117; doi:10.1002/advs.202306862)
Supplement: Supplementary file 1 — Supporting Information [file ADVS-10-2306862-s004.pdf]

ADVANCED SCIENCE
Open Access

Supporting Information

for *Adv. Sci.*, DOI 10.1002/advs.202306862

Toward Tunable Protein-Driven Hydrogel Lens

*Maria Kaeek and Luai R. Khoury**

Toward Tunable Protein-Driven Hydrogel Lens

Maria Kaeek[1], *Luai R. Khoury*[1*]

[1] Department of Materials Science and Engineering, Technion Israel Institute of Technology, Haifa, 32000, Israel

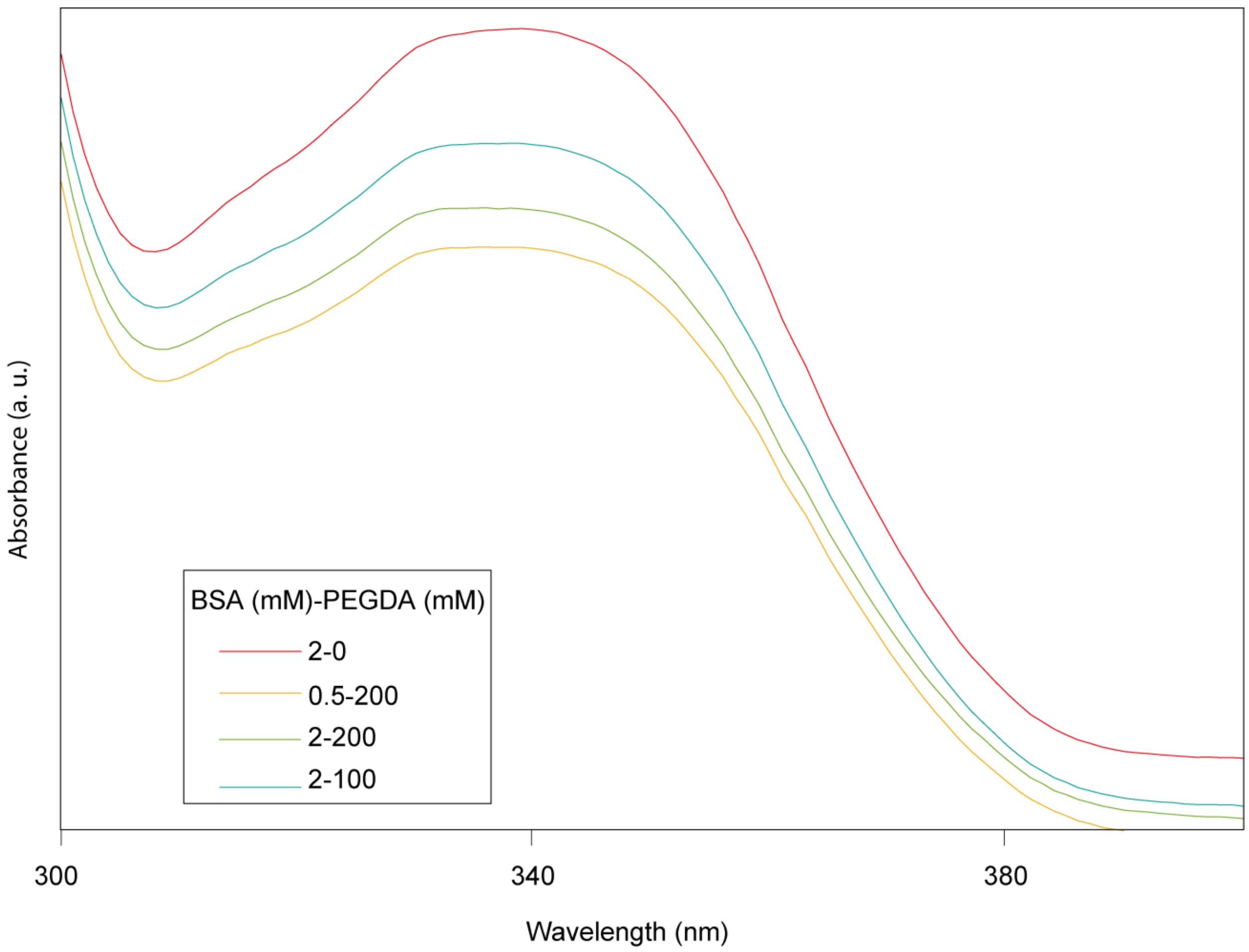

Supporting Figure 1. The effect of the concentration ratio of PEGDA and BSA on the degree of lysine residue functionalization. The UV-vis spectra of BSA and BSA-PEGDA were analyzed at various concentration ratios after incubation and reaction with 2,4,6-trinitrobenzene sulfonate (TNBS) to evaluate the extent of functionalization of available lysine residues on the BSA surface by PEGDA acrylates. TNBS reacts with the free primary amines of BSA lysine, forming a compound that exhibits strong absorption at approximately 342 nm. By comparing the absorbance of BSA-PEGDA at different

ratios to that of native BSA, the percentage of lysine functionalization of BSA with PEGDA acrylates was calculated.

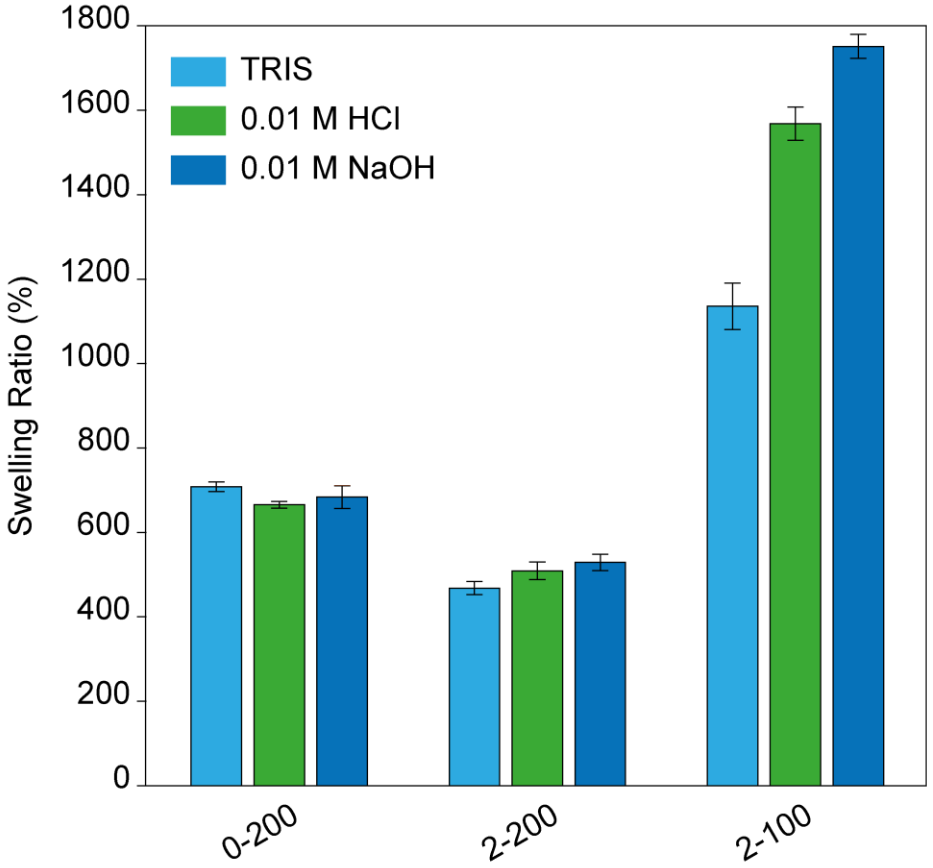

Supporting Figure 2. Effect of BSA-PEGDA Concentration Ratios, Acidic, and Basics Solutions on swelling behavior. Swelling ratio measurements of BSA-PEGDA-based hydrogels at different concentrations ratios (0-200 mM, 2-200 mM, and 2-100 mM) and in various experimental solutions. Error bars represent SD in all measurements.

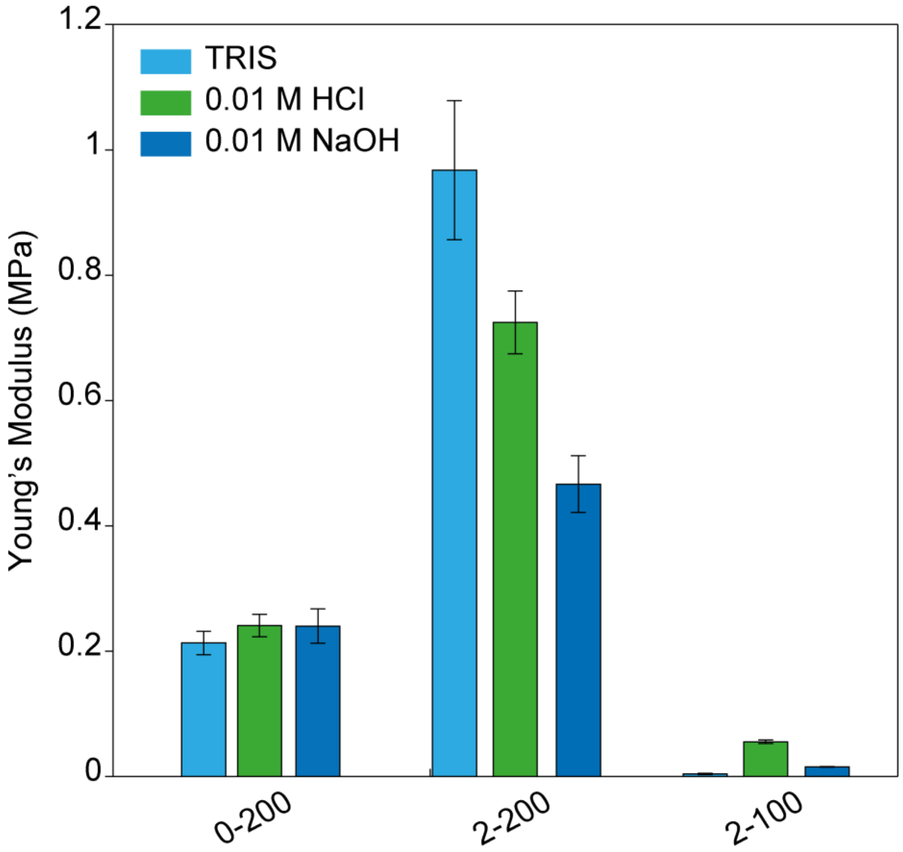

Supporting Figure 3. Effect of BSA-PEGDA Concentration Ratios, Acidic, and Basics Solutions on Young's Moduli of BSA-PEGDA-based Hydrogels. Average Young's moduli calculated from the compressive stress–strain curves of BSA-PEGDA-based hydrogels at different concentrations ratios (0-200 mM, 2-200 mM, and 2-100 mM). The samples were tested after being immersed in TRIS, 0.01 M HCl (pH=2), and 0.01 M NaOH (pH~12) for 1hr at RT. Error bars represent SD in all measurements.

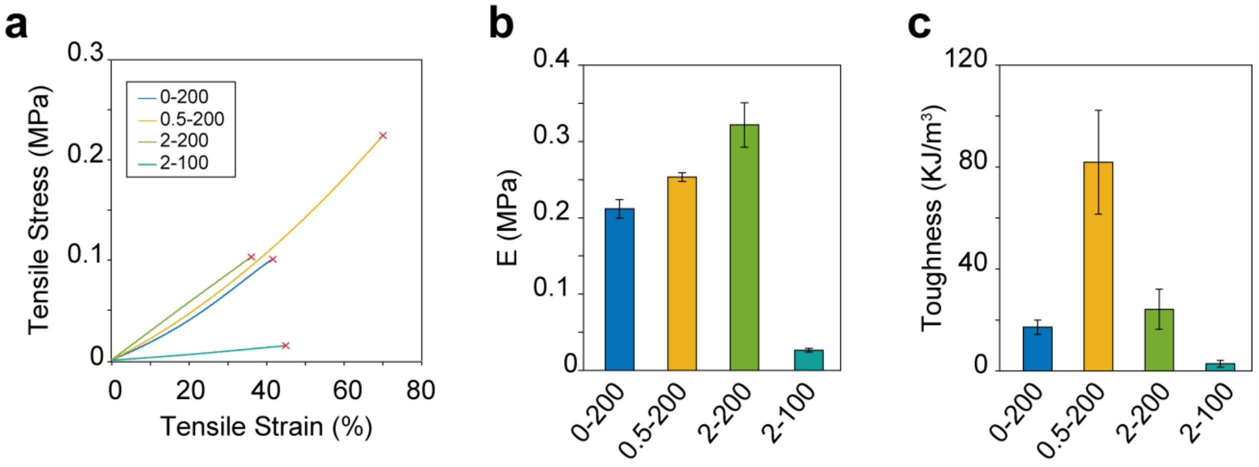

Supporting Figure 4. Mechanical characterization of BSA-PEGDA hydrogels at different concentration ratios under tensile tests. (a) Tensile stress – strain curves of BSA-PEGDA hydrogels. The tests were conducted with a tensile rate of 10 mm/min. (b) Average Young's moduli calculated from the tensile stress – strain curves of the BSA-PEGDA hydrogels. (c) Average toughness calculated from the area under the tensile stress – strain curves for the BSA-PEGDA hydrogels. Error bars represent SD in all measurements.

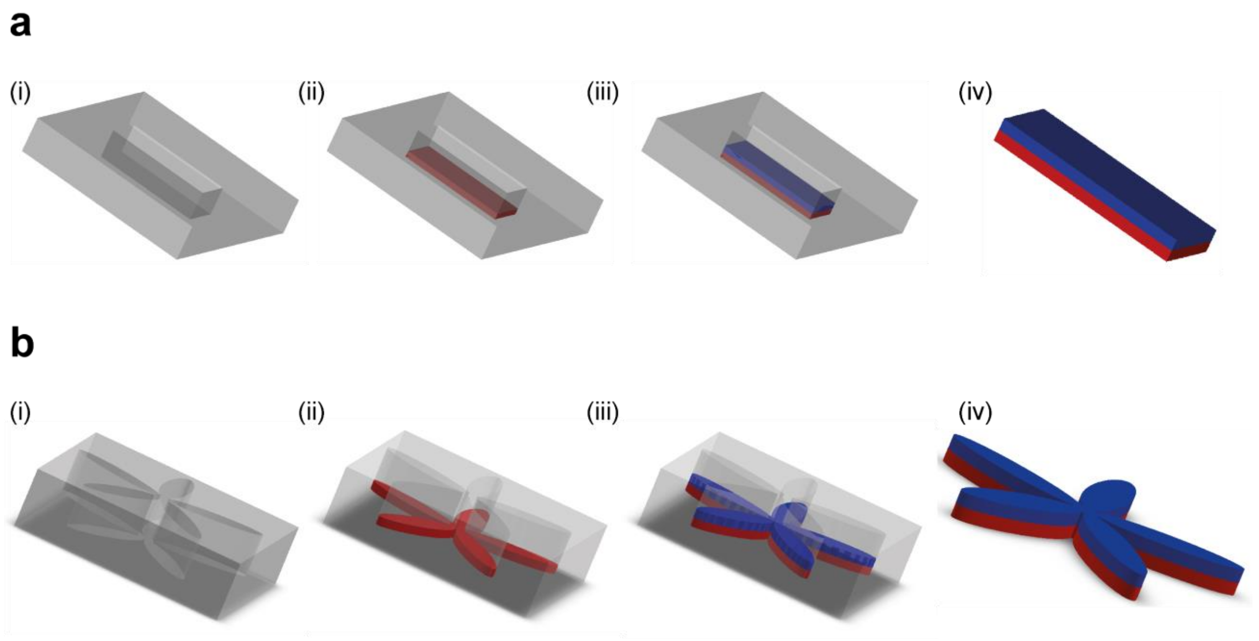

Supporting Figure 5. Fabricating Bilayer Hydrogel Systems. The fabrication process of the bilayer hydrogel system encompassed two distinct shapes: (a) bilayer rectangular hydrogel and (b) bilayer flower-shaped hydrogel. To achieve these shapes, 3D printing technology was employed to create the specific casts, which were filled with Dragon Skin™ silicones 20 to form the respective casting molds. The construction of the bilayer hydrogel system involved the following steps: (i) Extraction of the Dragon Skin casting molds from the templates. (ii) Casting a passive layer consisting of PEGDA into the molds, followed by UV curing. (iii) Sequentially, an active second layer composed of BSA-PEGDA-based hydrogel was added on top of the first layer and cured. (iv) Finally, the resulting hydrogel sample was extracted from the molds and immersed in TRIS solution at room temperature (RT) to attain equilibrium.

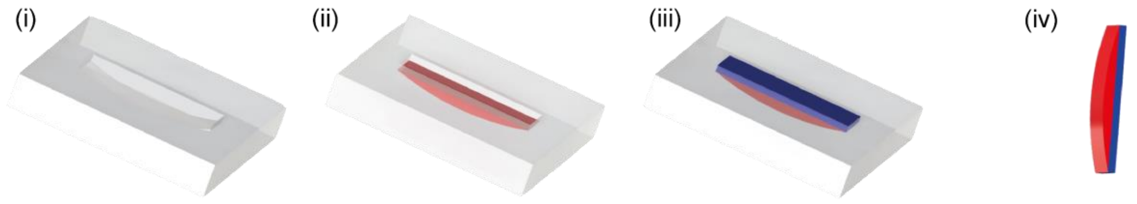

Supporting Figure 6. Fabricating Bilayer Protein-Driven Hydrogel Lens. The bilayer plano-convex lens system was manufactured using the following preparation steps: (i) The plano-convex lens cast was created using 3D printing technology, filled with Dragon Skin™ silicones 20 to form the lens casting molds. (ii) The spherical bottom part of the mold was filled with an active layer composed of BSA-PEGDA solution, followed by UV curing. (iii) After that, the passive second layer made of PEGDA was added on top of the first layer and cured to form the Plano layer. (iv) The resulting hydrogel lens was removed from the mold and placed in a TRIS solution at room temperature (RT) to equilibrate.

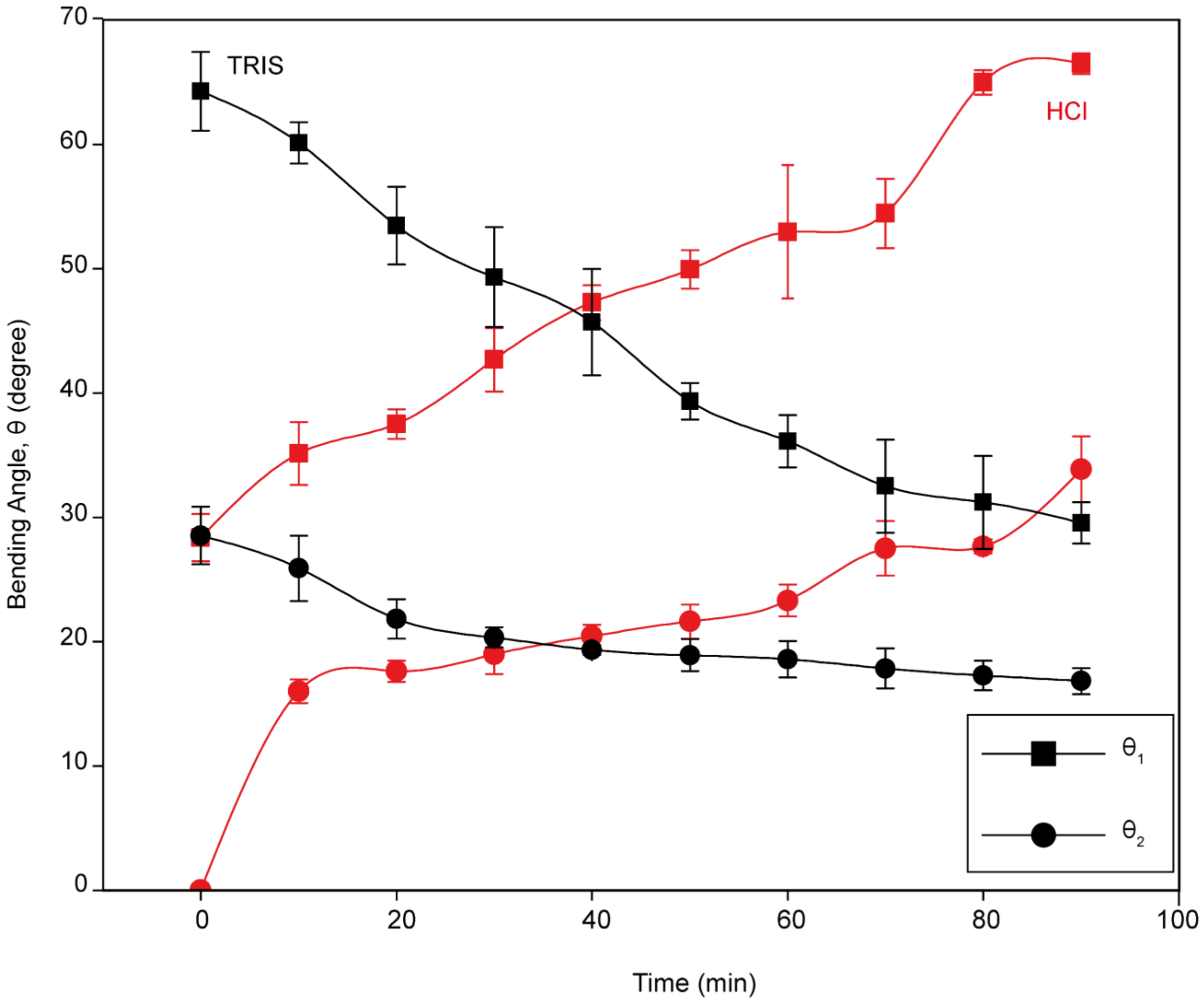

Supporting Figure 7. The morphing kinetics of BSA-PEGDA/PEGDA (2-100/0-200) hydrogel lens. The protein-driven hydrogel lens with the plano-convex shape was immersed in HCl for 1.5 hours. During this time, the lens underwent a shape-morphing process, transitioning from a plano-convex to a convex-concave shape. This transformation caused the lens to bend toward the passive layer, increasing both bending angles (red curves). Subsequently, the lens was returned to the TRIS solution for a similar duration (black curves), which decreased lens curvature. As a result, the bending angles decreased. Both bending angles θ_1 and θ_2 were measured every 10 min by analyzing the images extracted from the timelapse videos using ImageJ. Error bars represent standard deviations.

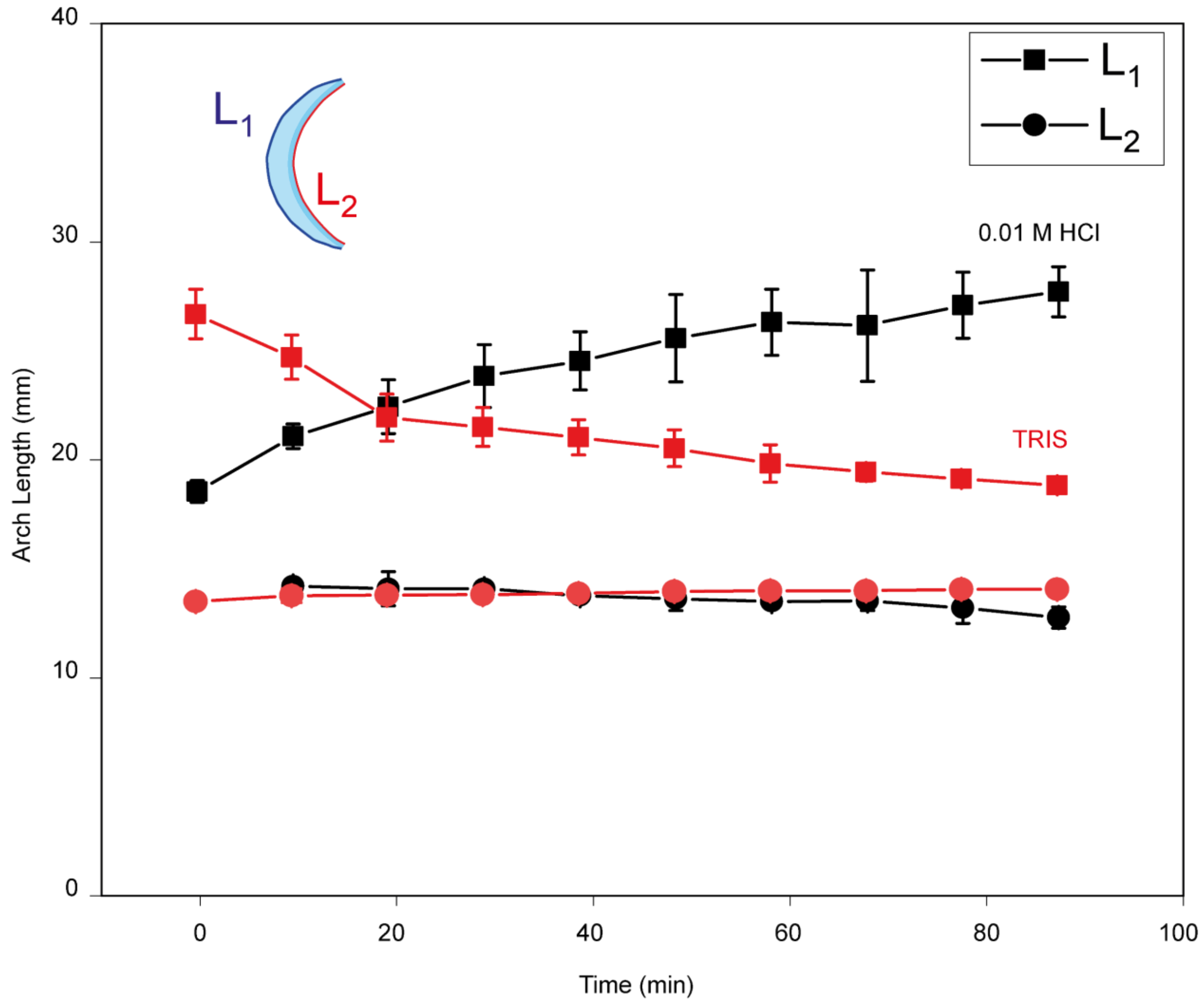

Supporting Figure 8. Arch length measurements of BSA-PEGDA/PEGDA (2-100/0-200) bilayer lens under different pH conditions. During the shape-morphing of the protein-driven lens from plano-convex to the concave-convex lens, the length of the outer arch of the active layer increased while the length of the inner arch of the passive layer slightly decreased (indicated by black lines) caused by the difference in swelling behavior between the layers. Subsequently, when the lens was soaked in TRIS solution, it induced the protein's refolding, allowing it to recover its initial shape and, in turn, resulted in the arches returning to their initial state (indicated by red lines). Both arch lengths, L_1 and L_2, were measured every 10 min by analyzing the images extracted from the timelapse videos using ImageJ.

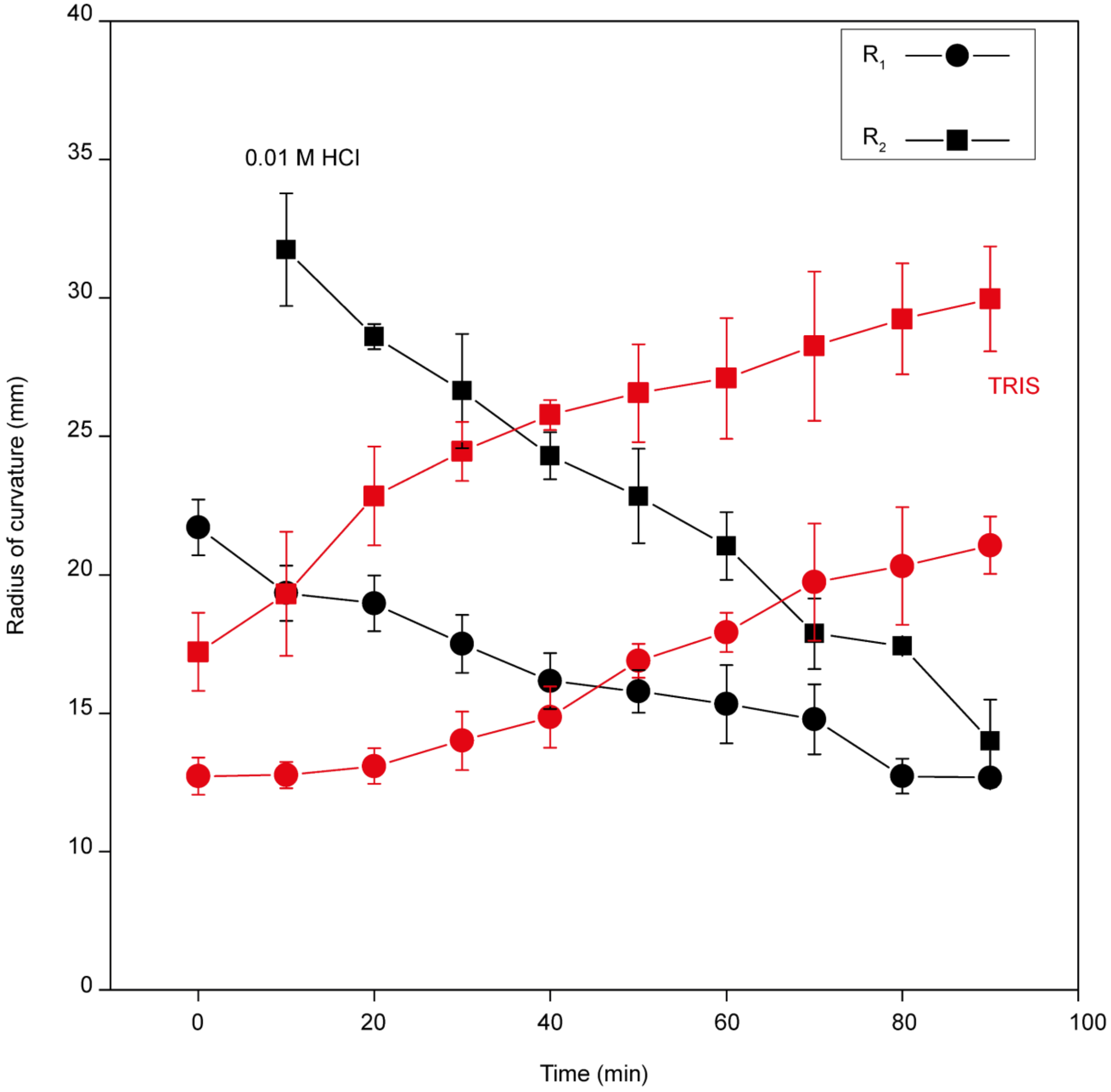

Supporting Figure 9. Radii of curvature of BSA-PEGDA/PEGDA (2-100/0-200) hydrogel lens under different pH conditions as a function of time. The radii of curvature, R_1, and R_2, also changed and calculated every 10 min based on the measured bending angles θ_1 and θ_2, the corresponding arch lengths L_1 and L_2, using the following equation $R_{1/2} = \frac{180^{\circ}}{\pi}\frac{L_{1/2}}{2\,\theta_{1/2}}$. Error bars represent standard deviations.

Supporting Movie 1. Acid-Induced Bilayer Hydrogel Deformation and Shape Recovery. A bilayer hydrogel sample was immersed in 0.01 M HCl (pH=2) for 1 hour at room temperature (RT). The recorded movie demonstrates that submerging the bilayer in an acidic environment induces the unfolding of BSA in the active layer, resulting in the bilayer bending towards the passive layer composed of PEGDA. The deformation becomes evident approximately 10 minutes after immersion in HCl (pH=2) and gradually reaches a maximum bending angle of approximately 120º after 1 hour. The variation in the bending angle is attributed to the dissimilarities in the swelling ratio and stiffness of each layer under the two conditions. The 0.01 M HCl (pH=2) solution was replaced with TRIS to observe the shape recovery process. Consequently, the bilayers gradually returned to their linear configuration in TRIS as the BSA molecules refolded in the active layer to their native state. The final recovered angle of the hydrogel in TRIS was approximately 20º. The progression was recorded using a time-lapse video, capturing one picture every 5 seconds.

Supporting Movie 2. Shape-changing Behavior of Bilayer Flower-shaped Hydrogels in Acidic and Neutral Environments. A flower-shaped hydrogel bilayer was immersed in 0.01 M HCl (pH=2) for 1 hour at room temperature (RT). The immersion of the bilayer in HCl prompted the closure of the flower petals, causing them to bend towards the PEGDA layer. Following the 1-hour HCl immersion, the hydrogel with closed petals was immersed in TRIS for an additional 1 hour at RT. In the TRIS solution, the flower-shaped hydrogel opened its petals. The entire process was recorded using a time-lapse video, capturing one picture every 5 seconds using top-view and side-view cameras.

Supporting Movie 3. Tunable Bilayer Protein-Driven Hydrogel Lens: Shape-Morphign and Focal Length Adjustment in Acidic and Neutral Environments. In the video, a bilayer hydrogel lens with a plano-convex shape (2-100/0-200 mM) is positioned perpendicular to a parallel dual-beam red laser box. Initially, the lens is immersed in a TRIS buffer and focuses the light at a focal length of approximately f=63±2 mm. Subsequently, the TRIS buffer is replaced with 0.01 M HCl, leading to structural changes in

the BSA protein and an increase in the swelling ratio and lens curvature. As a result, the lens transforms from a plano-convex shape to a convex-concave shape. This causes the focal length to gradually decrease to approximately f=23±2 mm as the lens bends. After around 90 minutes, the HCl buffer is exchanged with the TRIS buffer. Over time, the hydrogel lens regains its original shape, and the lens curvature decreases as the BSA molecules refold, increasing the focal length up to 44±3 mm. The process of adjusting the focal length of the hydrogel lens is recorded in a time-lapse video, capturing an image every 5 seconds.
